# The changing comet

**DOI:** 10.1186/s10194-017-0772-8

**Published:** 2017-06-28

**Authors:** Paolo Martelletti

**Affiliations:** Department of Clinical and Molecular Medicine, Sapienza University, Rome, Italy

Our usual virtual June meeting has arrived. This year we can see a number of positive trends: more and higher quality submissions, an explosion in the downloaded paper numbers, hyperactive social media, and finally a slight but significant increase in the Impact Factor.

Since 2008 the Impact Factor has doubled, and is now the highest it has been since the journal launched: 3.580. We are now in the first quartile of Clinical Neurology journals. The journal also demonstrated solid scientific relationships with other journals in the field, with citations both to and from a broad spectrum of journals.

We are still confident in the foundation of the journal: a historic act, driven by a desire to serve, rather than hubris. This can still be seen in the daily interaction between SpringerNature Editorial Office, and the affiliated scientific societies; favoring the speediness of interactions among authors and reviewers, and awarding ideas and quality.

The journal has never aimed to be an archetype, only a megaphone for every scientist involved in headache research. Our growth has surely benefited from a European history made by giants, but in time we have stepped down their shoulders with courage, despite some trepidation. Needless to say, being a whisker from the top is a pleasure mitigated only by the awareness that such positions, once reached, must be maintained or increased.

Our success doesn’t depend on single very highly-cited articles, and we don’t want it to [1]. We can now see the success of our approach, and the promise of an innovative product has completed its path. The comet-like distribution of scientific journals has changed [2]: the historic obstacles in the nucleus have melted, and the comet now contains a new resident, *The Journal of Headache and Pain*.
